# Symptom burden, mental health and quality of life among COVID-associated mucormycosis patients: A prospective cohort study

**DOI:** 10.6026/973206300212272

**Published:** 2025-08-31

**Authors:** Karanvir Singh Matharoo, Sushma Bhatnagar, Shoibam Jenifa, Rohan Chauhan, Santenna Chenchula

**Affiliations:** 1 Department of palliative medicine, Homi Bhabha Cancer Hospital and Research Centre, Visakhapatnam, Andhra Pradesh, India; 2 Department of Medicine, Clinical lead and senior consultant, Indraprastha Apollo Hospital, Sarita Vihar, New Delhi, India; 3 Department of Physical Medicine and Rehabilitation, All India Institute of Medical Sciences, Mangalagiri, Guntur, Andhra Pradesh, India; 4 Department of Medicine, Fortis Hospital, Ludhiana, India; 5 Department of Pharmacology, AIIMS Bhopal, India

**Keywords:** Mucormycosis, COVID-19, depression, anxiety, mental health, quality of life

## Abstract

COVID-associated mucormycosis (CAM) is a severe fungal infection with lasting physical and psychological effects. This prospective cohort of 53 CAM patients evaluated symptom burden, depression (PHQ-9), anxiety (GAD-7), and quality of life (WHOQOL-BREF) at baseline, 6 months and 1 year. Facial pain (89%) and headache (87%) were the most common symptoms; depression improved significantly (PHQ-9: 9.7 to 5.4; p < 0.001), while anxiety initially declined but rose again at 1 year. Quality of life improved in physical and psychological domains, whereas social and environmental aspects remained unchanged. Persistent challenges such as dysphagia (33%), voice changes (27%) and appearance concerns (27%) underscore the need for integrated long-term mental health and rehabilitation strategies in CAM care.

## Background:

Coronavirus disease (COVID) associated mucormycosis (CAM) emerged as a significant complication during the COVID-19 pandemic. Mucormycosis is a life-threatening, angioinvasive fungal infection caused by filamentous fungi of the order Mucorales. It predominantly affects individuals with underlying risk factors such as uncontrolled diabetes mellitus (DM), prolonged neutropenia and those receiving high-dose and/or long-term corticosteroid therapy [1]. Epidemiological studies indicate that the majority of mucormycosis cases have been reported in India [2]. In contrast to Western countries, where haematological malignancies and solid organ transplant recipients constitute the primary at-risk population [3, 4], uncontrolled DM is the leading predisposing factor in Indian cohorts. A recent nationwide multicentric study from India reported that 57% of patients with mucormycosis had uncontrolled DM, and 18% presented with diabetic ketoacidosis (DKA) [5]. Other Indian studies have similarly reported DM as a risk factor in 80-100% of cases [6, 7]. Effective management of mucormycosis relies on multiple factors, including prompt diagnosis, control of underlying comorbidities, timely surgical debridement of necrotic tissue, and administration of high-dose systemic antifungal therapy [8]. While much attention has been directed toward acute management, emerging evidence suggests that CAM survivors often face significant post-discharge challenges, including social, economic, emotional, and functional impairments [9]. Despite these observations, there remains a paucity of data on long-term clinical outcomes, quality of life, and the persistence of physical and psychological symptoms among CAM patients following hospital discharge. Therefore, it is of interest to describe the long-term symptom burden, mental health outcomes, and quality of life in CAM patients through a prospective cohort study.

## Methods:

## Study design and setting:

A prospective observational cohort study was conducted in the dedicated COVID-associated mucormycosis (CAM) ward at the All-India Institute of Medical Sciences (AIIMS), New Delhi, a tertiary care government hospital. Consecutive patients admitted between August 2021 and August 2022 were screened for eligibility. The study was approved by the Institutional Ethics Committee (Ref. No. IECPG-400/28.07.2021), and written informed consent was obtained from all participants prior to inclusion.

## Participants:

## Inclusion criteria:

Eligible participants were adults aged 18 years or older with microbiologically confirmed mucormycosis, identified via potassium hydroxide (KOH) microscopy, fungal culture, or histopathological examination. Radiological confirmation was required using contrast-enhanced MRI or CT of the affected anatomical region. A confirmed diagnosis of COVID-19, established by RT-PCR or rapid antigen testing within four weeks of presentation, was also necessary.

## Exclusion criteria:

Patients were excluded if they were unable to comprehend Hindi or English, exhibited an altered sensorium (Glasgow Coma Scale score <15) or cognitive impairment, or declined to provide informed consent.

## Data collection and instruments:

Data were collected using standardised tools at three time points: baseline (during hospital admission), 6 months, and 1 year post-enrolment.

## Symptom burden:

A semi-structured proforma captured the presence and duration of 15 predefined symptoms (*e.g.*, facial pain, vision loss).

## Anxiety:

The Generalised Anxiety Disorder 7-item (GAD-7) scale was used, with validated Hindi and English versions.

## Depression:

The Patient Health Questionnaire 9-item (PHQ-9) scale, also available in validated Hindi and English versions, was utilized.

## Quality of life:

The World Health Organisation Quality of Life-BREF (WHOQOL-BREF) instrument assessed four domains: physical health, psychological well-being, social relationships, and environmental factors.

## Demographic and clinical variables:

Data included age, sex, comorbidities, COVID-19 disease course, anatomical site of mucormycosis involvement, surgical interventions (*e.g.*, debridement, maxillectomy, orbital exenteration), and antifungal therapy.

## Follow-Up protocol:

Baseline assessments were conducted by trained investigators during hospitalisation. Follow-up evaluations were carried out at 6 months and 1 year, either through in-person interviews in the outpatient department or structured telephone interviews for non-ambulatory patients. Mortality data were verified using hospital records and, when necessary, by contacting the patient's next of kin.

## Statistical analysis:

Data analysis was performed using SPSS version 25.0 (IBM Corp.) and R version 4.1.2. Descriptive statistics were reported as mean ± standard deviation (SD) for normally distributed continuous variables, median [interquartile range] for non-normally distributed continuous variables, and frequencies with percentages for categorical variables. Normality of data was assessed using the Shapiro-Wilk test. Longitudinal changes in GAD-7, PHQ-9, and WHOQOL-BREF scores were analysed using linear mixed-effects models with subject-level random effects. Post hoc pairwise comparisons were adjusted using the Bonferroni correction. Secondary analyses employed Fisher's exact test for categorical variables and the Mann-Whitney U test or Kruskal-Wallis test for non-normally distributed continuous variables. Missing data were handled using multiple imputation based on a fully conditional specification. Statistical significance was defined as a two-tailed p < 0.05. Effect sizes were reported as Cohen's d for continuous outcomes and Cramer's V for categorical outcomes.

## Sample size:

Based on sensitivity analysis of the PHQ-9 scale (α = 0.05, power = 80%), a sample size of 45 patients was calculated to detect a 3-point difference in mean scores (standard deviation = 5.0). Accounting for an estimated attrition rate of 15%, a total of 53 participants were enrolled in the study.

## Results:

A total of 53 patients with confirmed COVID-associated mucormycosis (CAM) were enrolled in the study, with a mean age of 50.3 ± 11.7 years; 64% (n = 34) were male. All patients had diabetes mellitus, of which 74% (n = 39) had pre-existing diabetes and 26% (n = 14) were newly diagnosed during the index admission. Hypertension was the most common comorbidity (43%, n = 23). Anatomical patterns of mucormycosis involvement included sinonasal (47%, n = 25) and rhino-orbital (47%, n = 25) forms, with rhino-orbito-cerebral involvement observed in only 6% (n = 3) of cases. Surgical management was undertaken in all patients, comprising endoscopic debridement (48%), open maxillectomy (29%), and orbital exenteration (23%). The overall mortality rate was 15% (n = 8), with 75% (n = 6) of deaths occurring during the initial hospitalisation. The highest case fatality was noted in patients with rhino-orbital disease involvement (Table 1 - see PDF).

Facial pain (89%, n = 47) and headache (87%, n = 46) were the most frequently reported presenting symptoms. Ocular manifestations were also common and included periorbital swelling (70%, n = 37), blurred vision (53%, n = 28), and ophthalmoplegia (45%, n = 24). Other symptoms, such as nasal discharge (42%, n = 22) and facial swelling (60%, n = 32), were prevalent. The mean symptom durations ranged from 4.8 to 7.6 days, indicating an acute and rapidly progressive disease course (Table 2 - see PDF). Mean depression scores significantly decreased from baseline (9.7 ± 4.6) to the 1-year follow-up (5.4 ± 7.1; p < 0.001; Cohen's d = 0.74). The proportion of patients with moderate-to-severe depression declined from 49% (n = 26) at baseline to 13% (n = 6) at 1 year (p < 0.001). Concurrently, the percentage of participants reporting minimal or no depression increased from 9% (n = 5) to 64% (n = 29) (Table 3 - see PDF). Anxiety scores demonstrated an initial reduction at the 6-month follow-up (2.4 ± 2.2) compared to baseline (3.8 ± 2.8; p = 0.001; d = 0.54), but this improvement was not sustained at 1 year (2.9 ± 4.2; p = 0.10 vs. baseline). Throughout the follow-up period, the majority of patients experienced minimal to mild anxiety (82%-89%), though severe anxiety was reported in 2% (n = 1) at the 1-year mark (Table 4 - see PDF). Substantial improvements were observed in certain quality of life domains. The physical health domain demonstrated a significant increase from baseline (42.7 ± 13.7) to 1 year (57.6 ± 20.2; p < 0.001; d = 0.85). Psychological domain scores also improved (40.8 ± 16.2 to 48.8 ± 24.4; p = 0.002; d = 0.38). However, no statistically significant changes were observed in the social relationships domain (46.9 ± 14.1 to 50.7 ± 15.1; p = 0.43) or the environmental domain (51.6 ± 10.2 to 54.0 ± 11.1; p = 0.53) (Table 5 - see PDF). At the 1-year follow-up, several functional and psychosocial challenges persisted among CAM survivors. Functional impairments included dysphagia (33%, n = 15), voice changes (27%, n = 12), and issues related to poorly fitting prostheses (24%, n = 11). Psychosocial sequelae were also notable, with 27% (n = 12) reporting appearance-related concerns and 9% (n = 4) experiencing loss of employment. Of the 53 participants enrolled at baseline, 46 (87%) completed the 6-month follow-up, and 45 (85%) completed the 1-year assessment. Attrition was primarily due to mortality (n = 8) and loss to follow-up (n = 2). Sensitivity analyses using multiple imputation techniques confirmed the robustness of the primary findings.

## Discussion:

This prospective cohort study demonstrates that COVID-associated mucormycosis (CAM) is associated with substantial multi-dimensional morbidity. The condition was characterised by a notable mortality rate of 15%, a high symptom burden, including facial pain (89%) and headache (87%) and significant long-term psychological sequelae. Interestingly, the anatomical pattern in our cohort was dominated by rhino-orbital and sinonasal involvement (94%), which differs from the rhino-orbito-cerebral predominance reported by Ghosh *et al.* [10]. This contrast may be attributable to earlier diagnosis during the Delta variant-driven surge in India, allowing for timely clinical intervention before cerebral extension. The universal presence of diabetes mellitus in our cohort (100%) is consistent with previous national studies [8, 11, 12] and systematic reviews [13, 14], reinforcing uncontrolled diabetes as the paramount risk factor and highlighting the urgent need to prioritise glycaemic control as a key strategy in the prevention of CAM. Our longitudinal analysis revealed important insights into the mental health trajectories of CAM survivors. Depression symptoms, measured using the PHQ-9 scale, showed significant improvement over time, decreasing from a baseline mean of 9.7 to 5.4 at the one-year follow-up (p < 0.001). These findings are in line with the short-term improvements observed by Ahuja *et al.* [15], suggesting that depressive symptoms may be primarily reactive to the acute phase of illness and responsive to physical recovery. However, anxiety, as assessed by the GAD-7 scale, demonstrated a different trajectory. While scores initially decreased at six months, a rebound was noted at one year (2.9 vs. 2.4 at six months; p = 0.10 vs. baseline), indicating that anxiety may persist or resurface over time. This divergence points to the likelihood that anxiety is driven by ongoing challenges such as disfigurement (27%), dysphagia (33%) and job loss (9%). These findings underscore the importance of continued psychological support beyond the acute illness period, as recovery from CAM appears to be prolonged and complex.

Our study also evaluated longitudinal changes in quality of life using the WHOQOL-BREF instrument. Improvements were observed in the physical and psychological domains, which contrast with findings from Kumar *et al.* who reported sustained poor quality of life in CAM survivors [16]. Several factors may explain this discrepancy. A higher proportion of our patients underwent endoscopic debridement (48%) rather than radical surgery, potentially preserving function and expediting recovery. Cultural factors, such as strong familial support systems, may also have played a protective role in mitigating psychological stress. Additionally, our one-year follow-up period may have captured recovery trajectories missed in shorter-term studies. Despite these gains, scores in the social domain remained unchanged (46.9 to 50.7; p = 0.43), reflecting continued social stigma, isolation, and relational difficulties. This stagnation highlights the need for culturally sensitive psychosocial interventions that address social reintegration and stigma reduction. Beyond psychological outcomes, the study identified a substantial burden of functional impairments at one year post-treatment. Dysphagia (33%), voice changes (27%), and prosthetic complications (24%) were commonly reported. These sequelae highlight several critical gaps in post-discharge rehabilitation. Access to specialised speech and swallowing therapy remains limited, particularly for patients undergoing maxillectomy or orbital exenteration. Additionally, the high cost of maxillofacial prosthetics-often exceeding ₹25,000 (~$300)-represents a significant financial burden for many patients, potentially contributing to the observed psychosocial distress. Job loss, reported in 9% of participants, was frequently associated with visible facial disfigurement, suggesting the presence of workplace discrimination and societal exclusion.

This study has several limitations. As a single-centre investigation, the findings may reflect institutional practices and may not be generalizable to other settings. Moreover, attrition due to mortality and loss to follow-up (15%) may have led to an underestimation of long-term morbidity. The use of telephonic assessments, necessitated by pandemic-related constraints, may have limited the sensitivity of anxiety assessments, as in-person evaluations are generally preferred for tools such as the GAD-7. Additionally, the absence of a control group precluded comparison between COVID-related and mucormycosis-specific quality of life impairments. Nevertheless, these limitations were addressed, to some extent, through sensitivity analyses using multiple imputations, and the use of validated tools in Hindi enhanced cultural and linguistic appropriateness. Studies have demonstrated that booster vaccinations against SARS-CoV-2, including those targeting Omicron subvariants, significantly enhance protection and reduce clinical severity [17, 18 and 20]. Despite the success of vaccine programs, high-risk populations-including individuals with diabetes or obesity remain particularly vulnerable to severe COVID-19 and its complications, such as CAM [19]. These findings align with our cohort's profile, in which all patients had diabetes, reinforcing the need for targeted prevention strategies, including aggressive vaccination efforts in high-risk groups. Moreover, the mental health impact of the pandemic extended to healthcare providers, with recent data showing significant psychological and physical strain due to prolonged use of personal protective equipment (PPE) [21]. Such systemic stress may also influence care delivery and follow-up quality, particularly in resource-constrained settings. Epidemiological studies from India have further emphasised the diverse clinical presentations and evolving treatment outcomes during successive COVID-19 waves, which may also affect mucormycosis recognition and management [22]. Together, these emerging data underscore the need to interpret CAM outcomes not only through a clinical lens but also within a broader public health, immunological, and psychological framework. Based on our findings, several recommendations emerge for clinical practice and future research. There is a clear need to integrate routine mental health screening into post-CAM care, particularly for anxiety, using tools such as the GAD-7 at six and twelve months. Embedding psychologists into CAM follow-up clinics may facilitate timely intervention. Rehabilitation services must be expanded to include standardised protocols for swallow and voice therapy, especially for post-surgical patients. Government-subsidised programs for maxillofacial prosthetics would significantly reduce financial strain. Future research should focus on validating disease-specific quality of life instruments such as the MQOL-36 [9], conducting multicentre studies to identify socioeconomic determinants of recovery, and investigating inflammatory biomarkers that may elucidate the pathophysiological links between CAM and chronic psychological distress.

## Conclusion:

COVID-associated mucormycosis (CAM) leads to significant long-term morbidity, especially among patients with diabetes and rhino-orbital involvement. While depression improved over time, persistent anxiety and functional impairments such as dysphagia, voice changes, and prosthetic issues continued to affect quality of life. Limited social recovery and disfigurement-related unemployment further underscore the need for integrated mental health care, rehabilitation, and supportive policies for CAM survivors.

## Ethical Approval:

The study was approved by the Institutional Ethics Committee (Ref. No. IECPG-400/28.07.2021), and written informed consent was obtained from all participants prior to inclusion.

## Funding:

None

## Author contributions:

KSM, SC: Study design, data acquisition, and drafting the manuscript; SB, KSM: Conceptual support, data interpretation, and critical revision; KSM, SJ: Data analysis, manuscript preparation, and final approval; RC, SC, SJ: Literature review and referencing; SB, RC: Data validation and statistical review; KSM, SC: Formatting, proofreading, and table/figure preparation. All authors have read and approved the final version of the manuscript.
